# Low-Power and Low-Cost Environmental IoT Electronic Nose Using Initial Action Period Measurements

**DOI:** 10.3390/s19143183

**Published:** 2019-07-19

**Authors:** Carlos J. García-Orellana, Miguel Macías-Macías, Horacio M. González-Velasco, Antonio García-Manso, Ramón Gallardo-Caballero

**Affiliations:** CAPI Research Group and Instituto de Computación Científica Avanzada (ICCAEx), Universidad de Extremadura, E-06006 Badajoz, Spain

**Keywords:** gas sensors, IoT, ESP8266, low power, low cost, initial action period

## Abstract

In this work, we present a complete hardware development and current consumption study of a portable electronic nose designed for the Internet-of-Things (IoT). Thanks to the technique of measuring in the initial action period, it can be reliably powered with a moderate-sized battery. The system is built around the well-known SoC (System on Chip) ESP8266EX, using low-cost electronics and standard sensors from Figaro’s TGS26xx series. This SoC, in addition to a powerful microcontroller, provides Wi-Fi connectivity, making it very suitable for IoT applications. The system also includes a precision analog-to-digital converter for the measurements and a charging module for the lithium battery. During its operation, the designed software takes measurements periodically, and keeps the microcontroller in deep-sleep state most of the time, storing several measurements before uploading them to the cloud. In the experiments and tests carried out, we have focused our work on the measurement and optimization of current consumption, with the aim of extending the battery life. The results show that taking measurements every 4 min and uploading data every five measurements, the battery of 750 mAh needs to be charged approximately once a month. Despite the fact that we have used a specific model of gas sensor, this methodology is quite generic and could be extended to other sensors with lower consumption, increasing very significantly the duration of the battery.

## 1. Introduction

Monitoring with gas sensors is a subject of special relevance at present, from air quality in wearables to hazardous gases in industrial plants. In all of the cases, renouncing the use of cables to power the devices and to transfer data has many advantages, introducing us to the field of the Wireless Sensor Networks (WSN) and the Internet-of-Things (IoT).

A Wireless Sensor Network comprises a set of sensor nodes that can measure ambient conditions, such as detecting air quality through gas sensors that send the measurements wirelessly to a base station [1]. Each sensor node includes a radio transceiver, a power supply, a processing unit, sensors and a power management circuitry. Currently, there is also the possibility of a node, through the Wi-Fi infrastructure, directly publishing the information in the cloud using some of the known IoT platforms, although it is not widely used in the context of WSNs.

In the case of gas sensors there are many factors to take into account when designing a node: the target gas, sensitivity, speed of response, cost, stability, durability, safety requirements and lifetime, that is, the operating time without needing to use any external intervention, such as battery replacement [2].

Considering aspects such as good sensitivity, quick response, low-cost, and long life, semiconductor sensors (MOX) stand out as the ideal candidates for wireless nodes with gas sensors [3]. However, despite these advantages, this technology is not exempt from certain weaknesses such as low selectivity, weak reproducibility [4] and high-power consumption, the latter being a very important problem in the practical development of battery-powered wireless nodes.

**Figure 1:**
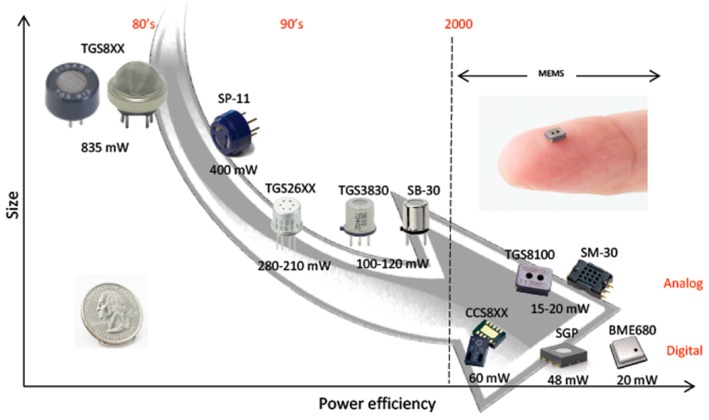
Reduction in the power consumption of MOX gas sensors in the last decades [3].

MOX sensors have a heating resistance to favour redox reactions on the sensing surface that is responsible for their high-power consumption. Although many efforts have been made to reduce the power consumption in MOX gas sensors (e.g., decreasing the size of the sensitive layer and of the heater area [5], thus reaching a power consumptions of tens of milliwatts, see Figure 1), there are still applications that need sensors with relatively high-power consumption to meet safety requirements [5,6,7].

Therefore, to operate with a MOX sensor, a supply voltage must be applied to the heater resistance, which consumes a significant quantity of energy. In practical applications, MOX sensors can operate in three different modes: continuously powered, periodically powered or on demand. When a MOX sensor is energized, it enters into an unstable state with a fall in the sensor’s resistance immediately after energization, followed by a transition period during which the initial stable resistance level is reached. This period is known as the *run-in phase* or *initial action* [8], and depends, among other factors, on the time the sensor is unenergized.

Likewise, operation in continuous mode consumes a lot of power and it is prohibitive in the design of wireless gas sensors nodes. In contrast, on-demand operation can cause the sensor to be unenergized for a long time and therefore the *initial action period* needed to take a new measurement can be very long and vary between consecutive measurements.

Apart from the physics of the sensors, there have been many studies that address the problem of reducing the energy consumption of the nodes, based on low energy communications protocols, architectures and solutions [9,10,11], and on the shape of the heating pulses applied to the sensors [3,12,13,14].

In the last case the more common technique is based on duty-cycling the activity of a node and its sensors. That is, the gas sensor is not polarized all the time, but only a small fraction of it and, evidently, the smaller that time, the greater the energy saving. On the other hand, and as we will see in the next section, sensor manufacturers do not recommend making this time shorter than the *initial action period*, what makes this methodology less efficient. In that sense, we presented in [14] a new technique to minimize this time, called *“gas sensor measures during the initial action period of duty-cycling”*. In that work we put the emphasis on demonstrating the validity of the presented technique, but we did not make any additional study on the power consumption when the new technique is used in the construction of a real wireless sensor node.

In this work we will present the hardware development and a power consumption study of a very low-cost gas sensor node that collects data and publishes them directly in the cloud using the *Thingspeak* IoT platform, through the Wi-Fi infrastructure. The designed low-power node uses the new technique described in [14] to reduce the energization time of the sensors to the maximum. The node is built around the System On Chip ESP8266EX, as a processing unit and Wi-Fi (IEEE802.11 b/g/n) transceiver, and a gas sensor from Figaro’s well-known TGS26xx family, since the new duty-cycling technique was validated with these kind of sensor.

The Methods used in the construction of the electronic nose, with special emphasis on the description of the correction technique proposed in [14], are described in Section 2. Results are shown in Section 3 and, finally, conclusions are presented in Section 4.

## 2. Methods

In this section, we will expose the most important techniques applied in the work, as well as the main hardware and software components that we have used to develop the proposed system.

### 2.1. Measuring in the Initial Action Period

There are many works in the literature related to power management and saving in systems that use MOX gas sensors, based on duty-cycling the activity of a node. In a duty-cycling operation, a node follows a sleep–wake–sample–compute–communicate cycle in which the longest part is the low-power sleep state [15]. This process reduces the average power consumption of a node by many orders of magnitude. The duty-cycle (*D*) of a node’s activity is defined as the fraction of time when the node is active [16], as shown in Equation (1).

(1) $$D=\frac{t_{active}}{T}\;\;\;\;\mathrm{where}\;\;\;\;T=t_{active}+t_{sleep}$$

Hence, to decrease the power consumption of a node, one has to reduce its duty-cycle *D*, and to reduce the duty-cycle, the active time $t_{active}$ should be decreased and the period *T* increased as much as possible. A duty-cycled operation is usually possible in WSNs since they are not required to sample or communicate continuously.

However, there are factors which prevent $t_{active}$ from being reduced as much as desired. One of those factors is the transient in the gas sensor’s response. When a stannic oxide sensor is energized after being stored under ambient conditions for a long time, two transients are observed prior to the final stabilization, a short-term transient which may last tens of seconds or even minutes, and is usually called the initial action transient, and a long-term transient which may last from a week to a month [8].

The short-term transient is characterized by a fall in the sensor’s resistance immediately after energization, followed by a transition period during which the initial stable resistance level is reached. When we use the measurement circuit recommended by the manufacturer, the sensor resistance is converted to a voltage through a voltage divider, and this voltage is considered the sensor response, which rises as the resistance falls. The duration of the initial action depends on the atmospheric conditions during storage and the length of storage, and varies with different sensor models [17]. So when a duty-cycling sensor is switched on, the sensor’s response rises sharply for the first few seconds after energizing, and then reaches a stable level in accordance with the ambient conditions (see Figure S1 in Supplementary Materials).

Therefore, when a node in a WSN is woken up and the sensor is newly energized, the $t_{active}$ of the node should be greater than the *initial action period* of the sensor, in order to have time to stabilize before measurements can be taken. Hence, the initial action of the gas sensors imposes a lower limit on how much the node’s power consumption can be reduced.

In [14], we proposed a correction for the sensor’s response in the *initial action period* so that measurements taken during this period could be useful. As a result, it is possible to reduce the value of $t_{active}$ of the nodes in the sensor network, and therefore their power consumption. In summary, measurements can be taken during this initial action powering-up time, and the initial action error can be corrected with our method, eliminating the short-term transient (the rise in the sensor’s responses immediately after energization).

The proposed method consists in carrying out a linear transformation of the datum measured after *n* seconds of the *initial action period* start, so that this transformation corrects the error with respect to the sensor that is permanently powered, which we consider as reference. In this way, a different transformation is obtained for each value of *n*, but we found that, with the TGS26xx series, the correction stabilize for n>50 s. Though the specific details of the process can be found in [14], the correction technique simply consists in applying Equations (2) and (3), where $S_{n}^{ref}$ and $S_{n}$ are the measurements of the reference sensor and the switched sensor respectively, taken *n* seconds after switching on, $a_{n}$ and $b_{n}$ are constants obtained by regression and are different for each value of *n*, while *a* and *b* are also constants obtained by regression, but once the stable value has been reached (in the case of TGS26xx, when n>50 s). Finally, $S_{n}^{c}$ is the corrected value of the measurement.

(2) $$S_{n}=\left\{\begin{array}{cc} a_{n}+b_{n}\cdot S_{n}^{ref} & \mathrm{for}\;n<50\;s\\ a+b\cdot S_{n}^{ref} & \mathrm{for}\;n\geq 50\;s \end{array}\right.$$

(3) $$S_{n}^{c}=a+b\cdot \frac{S_{n}-a_{n}}{b_{n}}$$

In that work, we also showed that there is a trade-off between the error (the square of the correlation coefficient value, $r^{2}$, obtained when we correlate the responses of the duty-cycling sensor and another sensor powered continuously) and the power savings (clearly dependent on *n*, the time in seconds after power-on). For n=1 s (maximum power savings), the $r^{2}$ value was 0.81, while for n=5 s, the $r^{2}$ value rose to 0.95. But for n=20 s, the $r^{2}$ value was 0.98, with the behaviour being similar to the case of correlating the two continuously powered sensors.

To reach these conclusions, we tested four different TGS gas sensors (TGS2600, TGS2602, TGS2611, and TGS2620) in [14]. Their results were similar, so for simplicity we only presented the results obtained with the TGS2600 in that paper. The three TGS2600 gas sensors were placed in a sensor chamber, and different gases (air, ethanol, tobacco smoke) were randomly passed through the chamber using a micro-pump, so that all the sensors were exposed to the same gas. Since the main objective was to study the correlation between the responses of the permanently energized sensor and the switched one, we didn’t consider relevant the concentrations and concrete composition of the gases to which the sensors were exposed, but only that the conditions of measurement varied. Measurements were taken every second, collecting 250,000 measurements (approximately 76 h) for each sensor, 2400 in each sleep–wake cycle (40 min).

**Figure 2:**
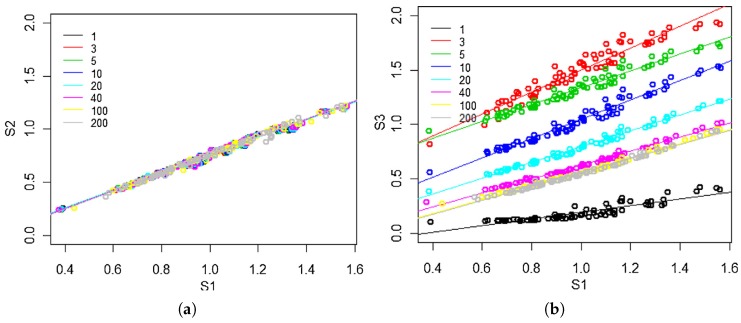
Correlations of data from three sensors of the same model. S1 and S2 are permanently powered, while S3 is the switched sensor. (**a**) S1 vs. S2 for n=1,3,5,10,20,40,100,200 s. (**b**) S1 vs. S3 for n=1,3,5,10,20,40,100,200 s. Taken from [14].

Figure 2 shows the result of the correlations for different values of *n*. In that figure, data from three sensors of the same model are shown, where S1 and S2 are permanently powered, while S3 is the switched sensor (whose response we want to correct). It can be observed in Figure 2a how the correlation between S1 and S2 is independent of *n* (which was to be expected, since they are permanently connected and are the same model). Besides, Figure 2b shows how the correlation depends on *n* in the case of S1 and S3, though from n=40 s the dependence practically disappears.

**Figure 3:**
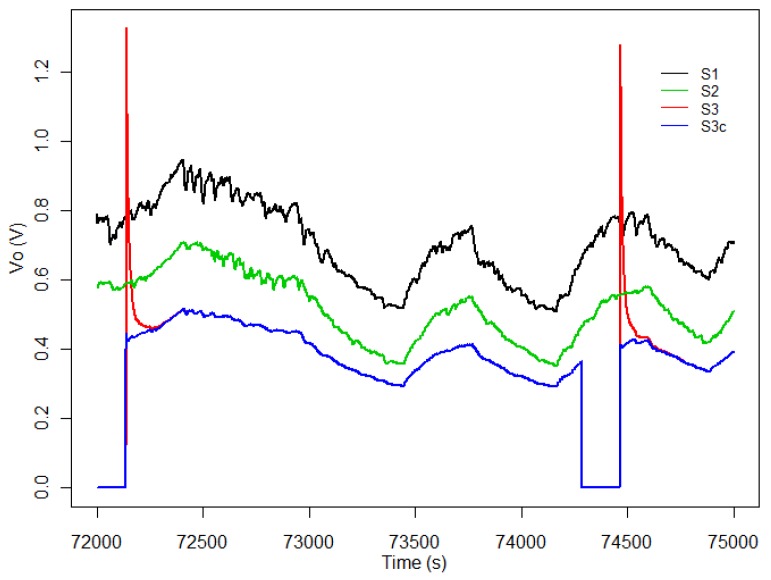
Response of the three sensors, for a period between 72,000 and 75,000 s, showing the effect of the application of the method described in [14].

On the other hand, Figure 3 shows the responses of the three sensors for a period between 72,000 and 75,000 s. As we can see in Figure 3 no drift correction method was applied over the sensors’ responses. The lines black and green correspond to the sensors powered continuously and the other one (red) corresponds to the duty-cycling sensor. In blue we can observe the corrected response of the the same sensor $S_{3n}^{c}$ in which the short term instability in the *initial action period* period has been corrected.

### 2.2. Main Components

Next, we will describe the main electronic components used in the development of the proposed IoT sensory system, as well as the software platforms used.

#### 2.2.1. SoC ESP8266

**Figure 4:**
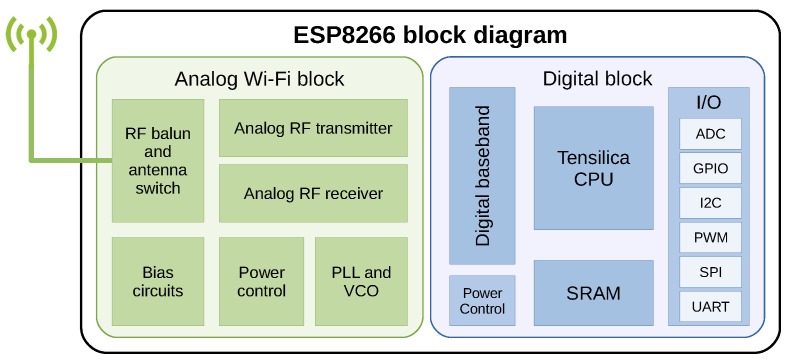
Block diagram of the ESP8266EX SoC.

The ESP8266EX [18] is a System On Chip (SoC) that combines a powerful microcontroller with a Wi-Fi transceiver, which makes it suitable for low-cost IoT applications due to its low price. This SoC is designed and manufactured by Espressif Systems (Shanghai, China), and it is a very popular device with many applications. In Figure 4 we can see a block diagram of the SoC. Regarding its technical characteristics, we can highlight the following:

- Tensilica 32 bits microcontroller, 80 or 160 MHz clock speed.
- 64 KB program memmory, and 96 KB for data.
- Power supply from 2.5 to 3.6 V.
- Wi-Fi transceiver with 802.11 b/g/n support.
- Low power consumption modes.
- Common peripheral interfaces (SPI, I2C, UART, etc...)
- One ADC (Analog Digital Converter) pin with 10-bit precision.
- OTA (Over the Air programming) support.

The ESP8266EX does not have flash memory to store the program to be executed. Therefore, it needs an external flash memory, which is connected by SPI bus. Because of this, we usually find the ESP8266EX as part of a module that, in addition to the aforementioned flash memory, includes the minimum necessary components and the Wi-Fi antenna. In this work we used the module known as ESP-12E, which is very popular, not only due to its characteristics, but also due to its price, since it has a cost of approximately 1.5 €. The ESP-12E module has everything needed for our application. In particular, we need a GPIO (General Purpose Input Output) for the control of the power supply which feeds the electronics accompanying the sensor, an ADC input to monitor the battery, and the I2C bus for the ADC that will measure the voltage obtained in the gas sensor.

##### Low Power Modes

For our work, one of the most important aspects of the ESP8266EX SoC are its low-power modes [19]. Specifically, the ESP8266EX has three main modes of low consumption, that we summarize in Table 1. Of these modes, the most relevant for us is the *Deep-Sleep*, since the SoC is completely asleep in this mode, except for the RTC (Real Time Clock). In order to wake up from this mode it is necessary to connect the terminals of RESET and GPIO16, since once the ’Deep-Sleep’ time has elapsed, the GPIO16 terminal will turn on and restart the SoC. The reason for the restart can be later ascertained by software.

The RTC is a clock that stays active even in the *Deep-Sleep* mode and allows to control how long the ES8266EX is in that energy saving mode. For our application, the existence of what is known as *RTC memory* is very important. The *RTC memory* is a 768 bytes RAM that maintains its value between the *Deep-Sleep* mode restarts. Of those 768 bytes, only 512 are for the user. This memory area can be used by our application to temporarily store a set of measurements before uploading them to the IoT server, which allows us an additional energy saving by not having to activate the Wi-Fi modem.

#### 2.2.2. TGS 2620 Gas Sensor

**Figure 5:**
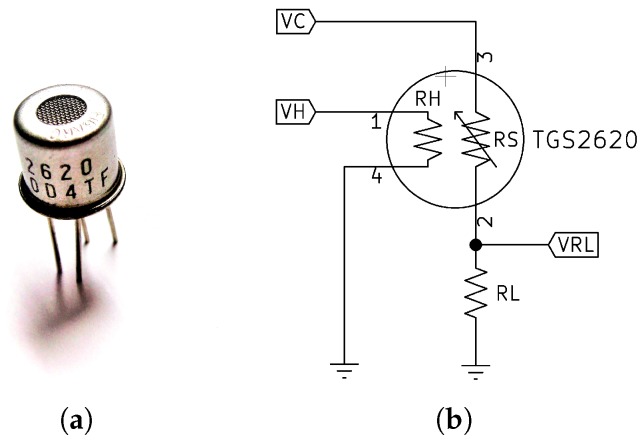
(**a**) A photograph of the TGS2620 sensor. (**b**) Measurement circuit recommended by the manufacturer [20]. It is important to maintain the polarity of the sensor element.

As we mentioned in the introduction, there are currently very-low-power sensors on the market, with heating resistors operating at 1.8 V, which are very interesting for low-power applications. However, in this work, we decided to use the TGS 2620 [20] sensor from Figaro Engineering, Inc. (Osaka, Japan), a sensor based on Metal Oxide Semiconductor (MOX), for several reasons. First, the Figaro’s TGS26xx series sensors are well known, studied and of proven quality. Besides, though its power consumption is higher than in other more recent sensors, we know from our previous work [14] that we can successfully apply the measurement method in the *initial action period* with the Figaro’s TGS26xx series, which significantly reduces the consumption. Nevertheless, our methodology and design does not depend on any specific sensor, and it could be adapted to any other MOX sensor provided that we can apply this measurement method. On the other hand, the TGS2620 (see Figure 5a) it is an adequate sensor for the detection of low levels of alcohol and organic volatiles [20], with an optimum detection range from 50 to 5000 ppm of concentration. It is currently used in many practical applications, such as in the tea flavor estimation [21], in the detection of volatile organic compounds [22] or in the monitoring of mint leaves during the drying process [23].

As it is usual in other MOX sensors, the TGS26xx series presents reproducibility problems and long term ageing, as well as important differences in behavior when replacing a sensor by another of the same model, making necessary a periodic recalibration in most cases [24]. In addition, they also have problems with short-term stability and reproducibility, although this can be partially corrected [25].

The TGS2620 heating element is powered with 5 V and consumes approximately 210 mW. The maximum power dissipated by the sensor element must be less than 15 mW. Figure 5b shows the measurement circuit recommended by the manufacturer, which emphasizes that the polarity of the sensor element indicated in the figure must be maintained, but not necessarily for the heating element.

Using the proposed circuit, the resistance of the sensor element ($R_{S}$) can be calculated according to Equation (4), while in order to know the maximum power dissipated by the sensor element ($P_{S}$) we can use the Equation (5), which also allows us to determine the minimum value of $R_{L}$.

(4) $$R_{S}=\frac{V_{C}-V_{RL}}{V_{RL}}\times R_{L}$$

(5) $$P_{S}=\frac{{\left(V_{C}-V_{RL}\right)}^{2}}{R_{S}}$$

Although the TGS2620 is considered a low consumption sensor, Figaro recommends a warm-up of at least 2 min, which makes any low-power application powered by small batteries unviable. For this reason, we decided to use the measurement technique in the *initial action period*, which reduces to a minimum the sensor consumption.

#### 2.2.3. Precision Analog to Digital Converter

To measure the voltage on the TGS2620 sensor we used a pull-down resistor and a precision ADC. We chose the ADS1100 model from Texas Instruments (Dallas, TX, USA) [26], which is a 16-bit converter with auto calibration, low-power consumption, programmable gain, wide power supply range (from 2.7 to 5.5 V) and I2C interface. The ADS1100 is designed for high resolution measurements, offering 8 SPS with 16-bit accuracy. It is presented in a SOT23-6 small package, and its current consumption when idle is 90 μA, although this fact is not very important in our case, since we will disconnect the ADC when not using it.

#### 2.2.4. ThingSpeak

ThingSpeak [27] is an important element of our work, since it is the IoT platform that we chose to upload the data to the cloud. The use of IoT in our system allows monitoring to be followed in any part of the globe with a simple web browser, and also lets us simplify the system by not having to store large amounts of data locally.

ThingSpeak started as an *open source* project in 2010 by ioBridge. It is an IoT platform, organized in *channels*, that provides a web interface (see Figure S2 in Supplementary Materials) to consult data stored by sensors in these channels, and to carry out some other actions based on that data. The platform has an API based on HTTP, so that the sensors can upload or recover data.

The project is now managed by MathWorks Inc. (Natick, MA, USA), who has included support for the use of MatLab in the analysis and visualization of data. This has caused that the open version of ThingSpeak has not been updated for several years. However, it is possible to install the open version and have a private IoT service.

### 2.3. Proposed Circuit

**Figure 6:**
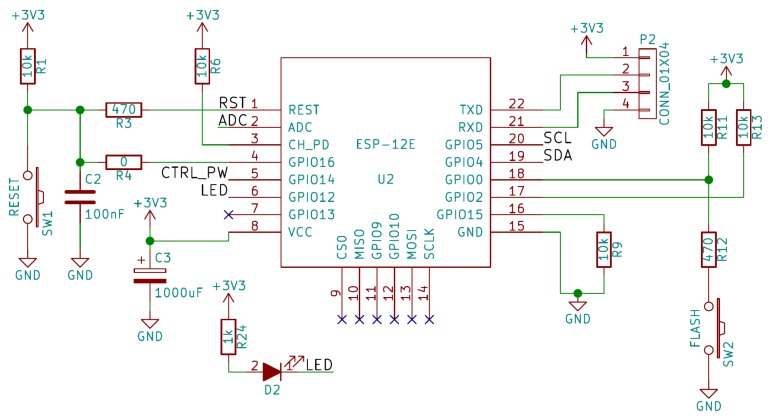
Schematic corresponding to the elements of the ESP-12E module.

**Figure 7:**
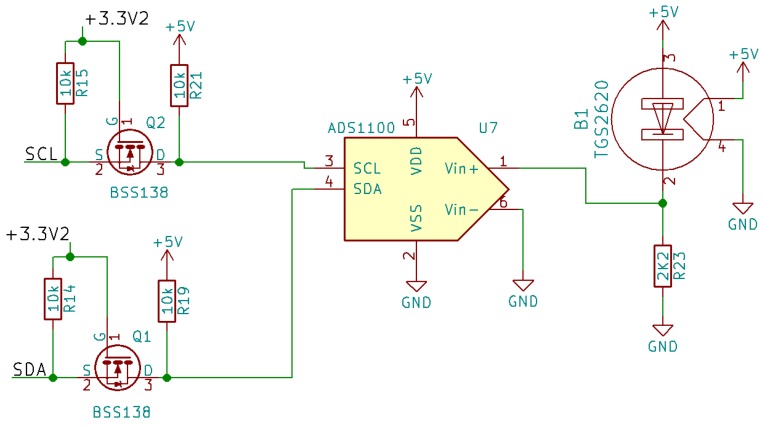
Schematic showing the TGS2620 sensor and the external ADC converter.

**Figure 8:**
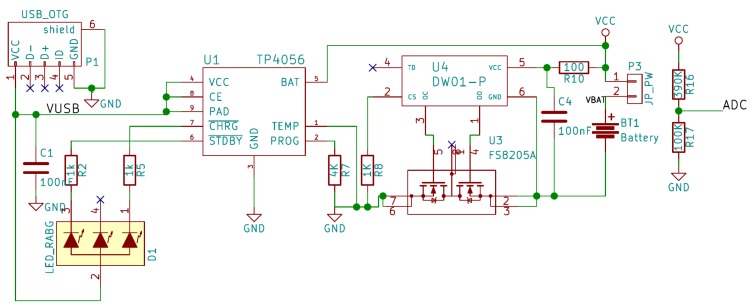
Schematic where the charging circuit of the battery that powers the design is shown.

**Figure 9:**
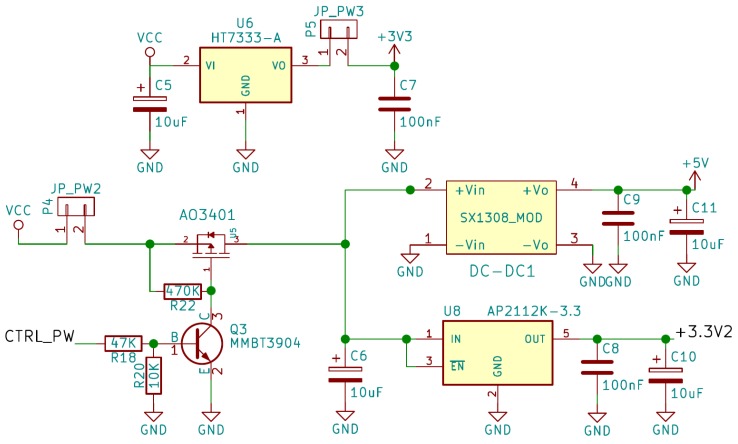
Schematic corresponding to the control and adjustment circuit of the power supply.

The circuit we propose to implement our IoT sensor can be seen in Figure 6, Figure 7, Figure 8 and Figure 9. As can be observed in these figures, the proposed system consists of four parts:

- Microcontroller (Figure 6).
- ADC and TGS2620 gas sensor (Figure 7).
- Battery charger (Figure 8).
- Power supply and its controller (Figure 9).

The first of the schematics corresponds to the circuitry necessary for the operation of the ESP-12E microcontroller module (Figure 6). This is the circuit generally used with this module, though we would like to highlight the presence of a large capacitor in the power supply, in order to be able to supply enough current in moments of high demand of the module when the battery is low. We would also like to highlight the presence of the R4 bridge to allow operation in *Deep-Sleep*. The GPIO14 was used to control the power supply of the rest of the design (CTRL_PW signal). Two buttons were included, SW1 for Reset and SW2 to put the ESP8266EX in programming mode. This circuit is permanently powered through +3V3.

Figure 7 shows the schematic corresponding to the TGS2620 gas sensor and the ADS1100 ADC converter. Since both the sensor and the ADC are powered with +5 V, we used level adapters to connect them to the microcontroller. The method we applied to measure with the TGS2620 sensor is proposed in its technical documentation [20], and has been discussed previously. This part of the circuit is powered only when the measurement is being made.

The next part of our system’s schematic is shown in Figure 8, and it is the charge and control circuit of the 3.7 V Lithium Ion battery, through a micro USB connector. The circuit consist of the chip TP4056, from NanJing Extension Microelectronics, to control the charging process, and a protection for the Li-Ion cell designed using the integrated circuits DW01-P and FS8205A from Fortune Semiconductor. In addition, a voltage divider (R16 and R17) is incorporated to measure the battery voltage through the internal ADC of the ESP8266EX. The charging current is controlled by resistor R7, and the status is indicated by an RGB LED. The jumper P3 (JP_PW) has been included to measure the charge and discharge current through the battery. The battery chosen in our case is a Li-Ion cell of 3.7 V and 750 mAh, and 14,500 size. The specific model selected for the system already includes a protection cell, so it could be eliminated from the design. However, we decided to keep it so that another battery without a protection cell can be chosen. The last important block of the proposed circuit can be seen in Figure 9, and it is the part that controls and adapts the power supply of the rest of the components. First, we have the HT7333 voltage regulator from Holtek Semiconductor (Hsinchu, Taiwan). It is a low drop-out 3.3 V regulator, and it is necessary because the ESP8266EX has a maximum supply voltage of 3.6 V and the fully charged battery supplies 4.2 V.

On the other hand, we can see in the lower part of Figure 9 the circuit responsible for connecting and disconnecting the power supply of the sensor and the precision ADC, in order to minimize consumption when the system is in sleep mode. The power supply connection and disconnection is carried out using a PMOS transistor (AO3401) with a low drain-source on resistance, using an output of the microcontroller control the transistor. To obtain the 5 V needed for the sensor we used a step-up DC-DC converter module, based on the SX1308 chip from SuoSemi Corporation (Shenzhen, China). We also included a second 3.3 V regulator to power the ADC level adapters only when necessary and thus minimize consumption.

### 2.4. Software Details

The software plays a very important role in the design of a low-power application. In our system we have considered two fundamental strategies to minimize the average consumption of the system and therefore maximize battery life. On the one hand, the measurement circuitry have been connected only when the system is measuring, and the Wi-Fi modem have been activated only when we are uploading data. Also, when the system is not in either of these two states (between measurements), we have kept the ESP8266 in *Deep-Sleep*. These states can be seen summarized in Table 2.

On the other hand, since the Wi-Fi connection is an important source of power consumption, we accumulated a certain number of measurements in the RTC memory area, uploading them to ThingSpeak in batches, in order to minimize the connection time.

**Figure 10:**
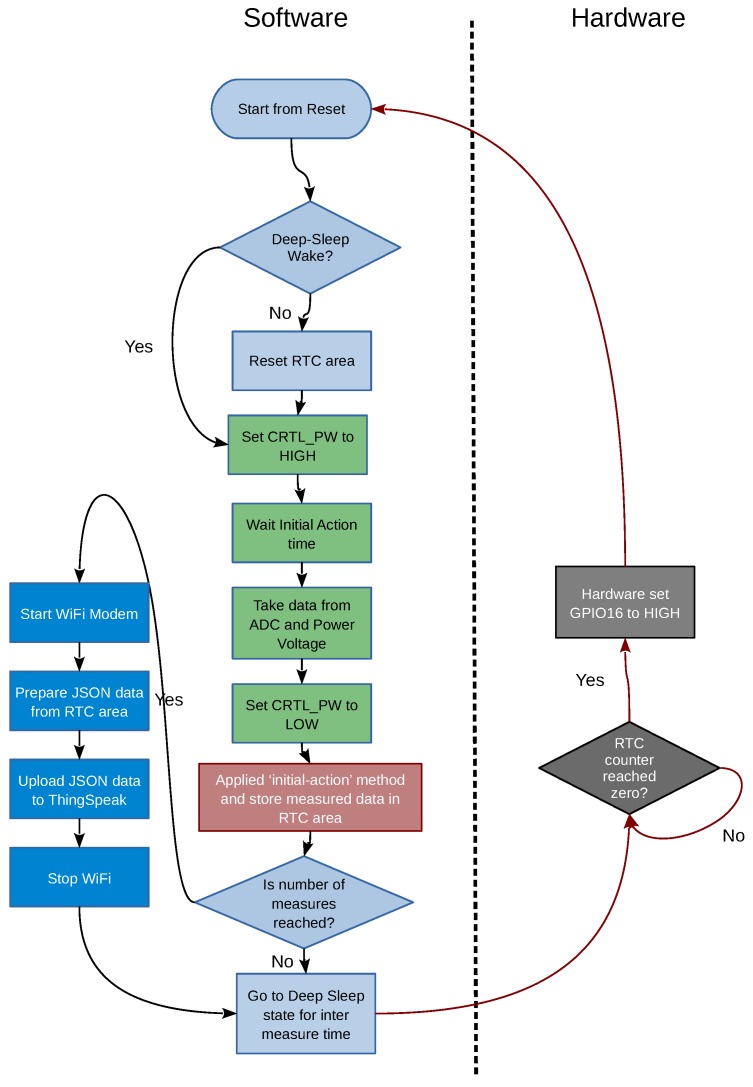
Diagram of the software developed.

In Figure 10 the diagram of the software implemented can be observed. In this figure we have separated the part of the software from the part of the hardware that the ESP8266 performs when it is in *Deep-Sleep* mode. First, the software checks the reason for the last restart. If it came from the *Deep-Sleep* state, a new measurement is made (otherwise the RTC memory area is initialized) and, if the number of measurements that form an upload batch ($N_{meas}$) has been reached, the Wi-Fi modem is activated and the batch of measurements (sensor data and battery status) is uploaded to the IoT ThingSpeak platform in JSON format.

As shown in Figure 10, the measurement process starts by activating the power supply of the TGS2620 and the ADC. Then, after waiting for a concrete time ($t_{meas}$), measurements are taken (from the sensor and the voltage supplied), and the power supply of the sensor and the ADC is turned off. Subsequently, the Equation (2) is applied to obtain the corrected value by using the measurement method based on the *initial action period*.

### 2.5. PCB and Assembly

For the assembly of the circuit in a compact way we designed a PCB (Printed Circuit Board), using SMD type components almost completely. The designed PCB is approximately 60 × 35 mm size, that is, quite smaller than a credit card.

**Figure 11:**
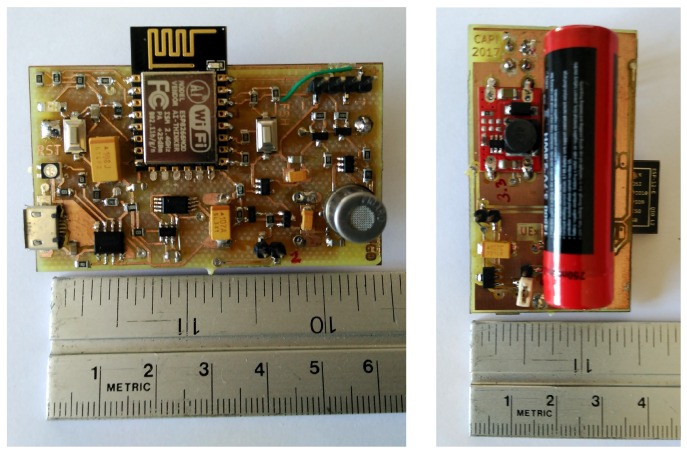
Image showing both sides of the final assembly.

The final assembly can be seen in Figure 11. In this figure, the dimensions of the assembly can be observed, as well as the layout of the components. On the upper side, the ESP-12E module can be seen, along with the push buttons, the TGS2620, the ADC ADS1100, the jumper JP_PW2, part of the power control circuit and the charging circuit with the micro USB connector. Also, the programming connector is placed in this side of the board.

On the back, the battery and the DC-DC step-up converter can be observed, apart from the main voltage regulator and two jumpers JP_PW and JP_PW3, which are used to measure the currents of the circuit.

To finish this section, we made an estimate of the total design cost. Depending on the chosen suppliers, the cost may vary a lot. By choosing the proper suppliers, we could build the proposed design for less than 20 €, including components and PCB, but not including assembly. We must bear in mind that only the TGS2620 can cost more than 20 € in some suppliers, so it is important to choose them well. Even in that case, the complete design did not exceed a cost of 40 € (without assembly).

The most expensive elements, apart from the sensor itself, are the Li-Ion battery and the ADS1100 converter (which could be replaced by the Microchip MCP3421, but at the cost of sacrificing performance a bit).

## 3. Results

In this work, we focus on the study of our design’s power consumption, comparing the different operating alternatives, and offering an estimate of the battery life. Therefore, we do not show any results of gas measurements, since the methodology of measurement in the *initial action period* has been validated in our previous work [14].

**Figure 12:**
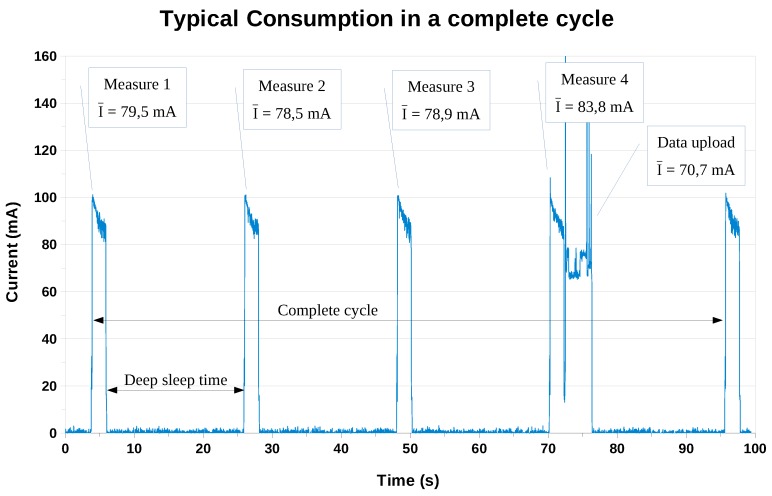
Typical evolution of the power consumption, considering a batch of 4 measurements and a rest period of 20 s.

As we explained in Section 2.1 and Section 2.4, our system’s operation is cyclical, alternating activity periods and deep-sleep periods. In our case, the periods of activity can be of two types: those in which we only take measurements, and those in which we upload a batch of data to the IoT platform after measuring. In Figure 12, the typical evolution of the consumption can be observed. In this particular case, we considered four measurements per uploaded batch ($N_{meas}$), a measurement time in the *initial action period* ($t_{meas}$) of 2 s, and a rest time ($t_{sleep}$) of 20 s.

As we stated above, our objective is to estimate the average power consumption in different scenarios. For this, we divided the study into three parts: “deep-sleep consumption”, “measuring-only consumption” and “measuring-and-uploading consumption” (Wi-Fi activated). With these base data, we studied the evolution of consumption in complete cycles, with different duration of each of the parts.

### 3.1. Study of Consumption in Each of the States

We next present the study of consumption in each of the three states mentioned above.

#### 3.1.1. Consumption in “Deep-Sleep” State

Most of the time our system will be ”waiting between measurements” (see Table 2), and in that state the ESP8266EX is in “deep-sleep” mode and the power supply of the measurement circuit is disconnected.

In this situation, the ESP8266EX must consume less than 20 μA, the charging circuit must consume less than 10 μA (according to its components datasheets), the HT7333 regulator 8 μA at most (according to its datasheet), and the voltage divider formed by R16 and R17, about 8 μA. This way, we expect a global consumption of about 46 μA, which we could reduce to 38 μA if the voltage divider were connected after the AO3401 transistor.

We made the consumption measurements in each of the three jumpers JP_PW, JP_PW2 and JP_PW3 (see Figure 8 and Figure 9) using an Amprobe 38XR-A multimeter (Amprobe GmbH, Glottertal, Germany), and the results are shown in Table 3. In this table, the errors shown correspond to twice the standard error calculated from 15 independent measurements. It can be observed a good correspondence with the expected consumption, as well as an extrapolation of the charge consumed for three different durations of deep-sleep mode. As is clear from the values, the amount of current consumed in this state is very small, especially if we compare it with the current consumed in the other states. However, given that the system will be in this state for a long time, it is important to consider this current. Equation (6) shows the charge consumed in this state ($CC_{sleep}$) as a function of the time (in seconds) that the system is in that state ($t_{sleep}$).

(6) $$CC_{sleep}\left(\mu As\right)=42.8\cdot t_{sleep}$$

#### 3.1.2. “Measuring-Only” Consumption

The measurement of consumption in the other states is somewhat more laborious. For this we used a precision clamp ammeter, of the brand Aim-TTi (TTI, United Kingdom), model I-Prober 520, which allows us to measure the instantaneous consumption with an oscilloscope (as it is shown in Figure 12).

In the state “measuring-only” we analysed numerous peaks for different values of the measurement time ($t_{meas}$). In this case we focused only on the global consumption (that is, on the jumper JP_PW), since it is what really interests us.

Specifically, for each measurement time of the *initial action period* ($t_{meas}$), about 10 to 20 peaks were analysed. From each of these peaks the average value of the current was obtained, and the global average was calculated for each value of $t_{meas}$. The results are shown in Table 4, in which we can find, for each value of $t_{meas}$, the average current, the average duration of the measurement process and the average consumed charge.

**Figure 13:**
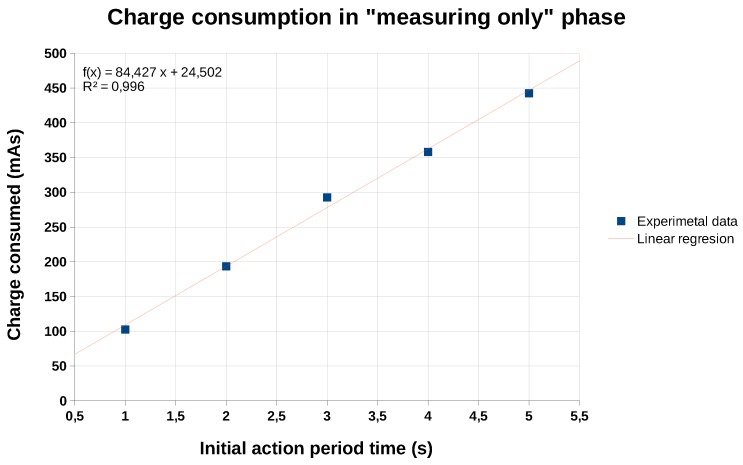
Charge consumed in the “measuring-only” state, as a function of the measurement time ($t_{meas}$).

In order to obtain an expression for the load consumed as a function of the measurement time ($t_{meas}$) we performed a linear regression using the data in Table 4. The graphical representation of this regression can be seen in Figure 13, in which we can observe a clearly linear trend of the charge consumption as a function of the measurement time. The correlation factor ($r^{2}$) is equal to 0.996 which gives us an idea of the accuracy of the adjustment. The expression in Equation (7) shows the value of the charge consumption, ($CC_{meas}$, in mAs), as a function of the measurement time used to implement the *initial action period* ($t_{meas}$, in seconds).

(7) $$CC_{meas}\left(mAs\right)=24.50+84.43\cdot t_{meas}$$

#### 3.1.3. “Measuring-and-Uploading” Consumption

The last state in which our system can work is found when, after the measurement, we must upload the data to the IoT platform. As can be seen in Figure 12, there are two clearly differentiated parts in this state regarding consumption: a first part that corresponds to the measurement (very similar to the state “measuring-only”) and a second part that corresponds to the data upload itself.

In this state the consumption of the part corresponding to the measurement is not exactly the same as in the case of “measuring-only” state. The reason is that, in this case, the Wi-Fi modem is available (although not activated yet) after the “deep-sleep” period, which causes the average current consumption to be a bit higher. However, this is offset by the fact that this measurement phase lasts somewhat less, as we go directly to the data upload process. This way, the charge consumed is practically the same as in the “measuring-only” state, so in order to simplify the calculations we will assume that the expression Equation (7) is valid in the first part of this state, since the error is less than 1% in almost all cases.

The charge consumption in the second part of this state, i.e., when we upload the data to the IoT platform, is much more variable than in the previous cases. This is because the connection and upload times are much more variable. Therefore, to obtain reliable statistics we analysed more than 75 data uploads and calculated the average current in each case, as well as the time. As a result, we obtained an average current of 70.8 ±0.3 mA and an average upload time of 3.10 ±0.07 s. Thus, we can conclude that the average charge consumption due to data upload ($CC_{upload}$) is 219.3 ±5.8 mAs. As in previous cases, the error shown corresponds to twice the standard error.

### 3.2. Global Consumption and Estimation of Battery Life

For the estimation of global consumption we must know when, and for how long, each of the partial consumptions acts. In a complete cycle (see Figure 12), the charge consumption will be given by the expression in Equation (8).

(8) $$CC_{cycle}=N_{meas}\cdot CC_{meas}\left(t_{meas}\right)+N_{meas}\cdot CC_{sleep}\left(t_{sleep}\right)+CC_{upload}$$

The number of cycles ($N_{cycles}$) that we can be performed with a battery of capacity $Bat_{cap}$ is given by Equation (9).

(9) $$N_{cycles}=\frac{Bat_{cap}}{CC_{cycle}}$$

Logically, to estimate the duration of the battery ($t_{bat}$) we must multiply the cycle time ($t_{cycle}$) by the number of cycles ($N_{cycles}$) that can be performed with that battery. The cycle time is the sum of the individual times that the system is in each of the three states. Two of those times are obvious: the deep-sleep time ($t_{sleep}$) and the upload time ($t_{upload}$). However, the time used in the measurement is not exactly the product of the number of measurements per cycle by the measurement time, since as we can see in Table 4, the average measurement time takes about 0.45 s more than the measurement time of the *initial action period*. Therefore, the cycle time would be given by the expression in Equation (10), and the battery life in Equation (11).

(10) $$t_{cycle}=N_{meas}\cdot t_{sleep}+N_{meas}\cdot \left(t_{meas}+0.45\right)+t_{upload}$$

(11) $$t_{bat}=N_{cycles}\cdot t_{cycle}=\frac{Bat_{cap}}{CC_{cycle}}\cdot \left(N_{meas}\cdot \left(t_{sleep}+t_{meas}+0.45\right)+t_{upload}\right)$$

Substituting the previous equations in Equation (11), and using the numerical values, we obtain the final expression shown in Equation (12).

(12) $$\begin{array}{c} t_{bat}=\frac{Bat_{cap}\cdot \left(N_{meas}\cdot \left(t_{sleep}+t_{meas}+0.45\right)+t_{upload}\right)}{N_{meas}\cdot \left(CC_{meas}+CC_{sleep}\right)+CC_{upload}}\Rightarrow \\ \Rightarrow t_{bat}=\frac{Bat_{cap}\cdot \left(N_{meas}\cdot \left(t_{sleep}+t_{meas}+0.45\right)+3.10\right)}{N_{meas}\cdot \left(24.50+84.43\cdot t_{meas}+0.0428\cdot t_{sleep}\right)+219.3} \end{array}$$

As we can see, the battery life is a function of the three fundamental parameters of the method used ($N_{meas}$, $t_{meas}$ and $t_{sleep}$). In Table 5 we can observe the estimated battery life for some combinations of these parameters that we think are more likely, assuming a battery capacity of 750 mAh.

**Figure 14:**
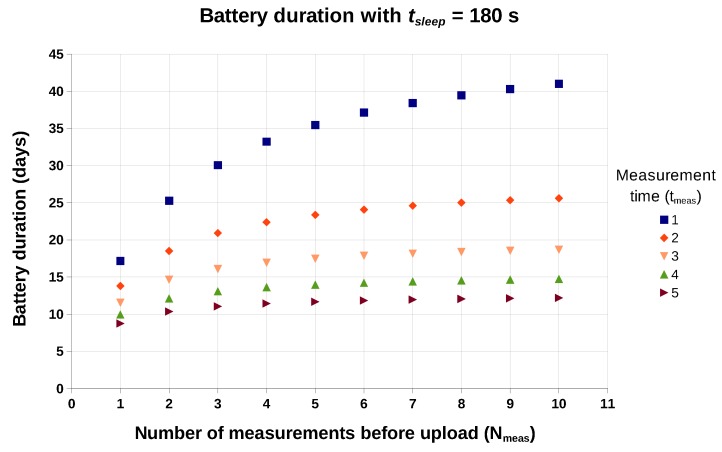
Estimated duration of a 750 mAh battery, keeping the deep-sleep time between measurements ($t_{sleep}$) equal to 180 s, as a function of $N_{meas}$ and $t_{meas}$.

**Figure 15:**
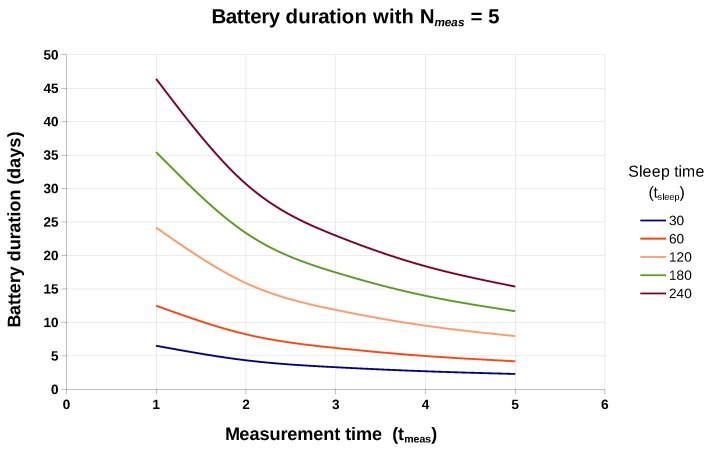
Estimated duration of a 750 mAh battery, keeping the number of measurements between uploads ($N_{meas}$) equal to 5, as a function of $t_{meas}$ and $t_{sleep}$.

It is also interesting to graphically see the evolution of battery life. Figure 14 shows the evolution of the battery life keeping the deep-sleep time fixed at 180 s. On the other hand, in Figure 15 we can observe this evolution when fixing the number of measurements between data uploads (5 measurements in the figure).

In Figure 14, we can see how increasing the number of measurements stored between data uploads ($N_{meas}$) improves battery life, but with an asymptotic behaviour (which would be easy to obtain by calculating the limit on Equation (12)). This way, from a certain number of stored measurements on, battery saving is not worth very much. Therefore, we could say that between 3 and 5 stored measurements would be adequate in most cases (except perhaps when the measurement time is 1 s).

On the other hand, we can see graphically in Figure 15 how the battery duration depends very sharply (especially for long deep-sleep times between measurements) on the duration of the *initial action period*. It is for this reason that, thanks to our methodology, we can consider building a system of this type powered by batteries.

Finally, if we analyse the results in Table 5 (along with the Figures mentioned above), it can be observed that in many cases a battery life of more than 15 days is achieved, provided that the deep-sleep time between measurements ($t_{sleep}$) is equal to or greater than 120 s, and the measurement time of the *initial action period* is at most 3 s. Specifically, if we consider a 750 mAh battery and take measurements every 240 s, with a measurement time of 2 s and uploading data every five measurements, we could obtain a battery life of 1 month.

With the previous data, the energy consumption per day would be about 90 mWh (considering a battery voltage of 3.7 V), an amount of energy that can be easily recovered with a small solar cell, and we would have a completely autonomous system.

To emphasize the importance of using the measurement in the first seconds of the *initial action period*, let’s consider the case in which we do not use the first seconds, but we follow the usual measurement procedure. In this scenario, we should let the sensor stabilize at least around 2 min [20] (i.e., we would have $t_{meas}=120$ s). Therefore, to have a measurement every 240 s, we should set $t_{sleep}$ in 120 s. Keeping $N_{meas}=5$, and applying Equation (12), we would have a battery duration of about 17 h. This means that using the measurement in the *initial action period* makes the battery last about 42 times longer.

If we calculate the energy consumption of this last case (waiting for the sensor to stabilize 2 min), we obtain 3.75 Wh. This way, to maintain the system with solar cells we would need a panel that gave an energy 42 times higher than the one needed when we use the measure in the *initial action period*.

Although the use of Wi-Fi is not common in the WSNs world, it avoids having to provide gateways and routing nodes to access IoT platforms, allows us to take advantage of an extensive indoor connectivity, and it significantly lowers the cost of the node. We mitigate the increase in consumption due to Wi-Fi by turning off the modem when not needed and accumulating measures to upload data to the IoT platform in batches.

We should also consider what would happen if we use an array of sensors instead of a single sensor. In this case, the correction in the *initial action period* would be made individually on each sensor, and then the corrected values would be used as input to a pattern classification system (formed, for example, by a feature extraction stage with PCA or ICA, and followed by a classifier based on SVM or neural networks). This classification could also be done in ThingSpeak, as MatLab is available on this platform. Regarding the consumption, we do not reckon that making the classifier’s inference calculation in the microcontroller would involve a significant power cost.

However, the use of several sensors would boost the consumption very significantly. For example, if we include a second sensor of the same type, the consumption of the new sensor heater would be, according to the manufacturer, about 210 mW. This power, with a voltage of around 3.7 V and a DC-DC converter efficiency of 90%, would result in an additional battery current of around 63 mA. If we consider the case in which the battery had a duration of one month ($t_{meas}=2$ s, $N_{meas}=5$ and $t_{sleep}=240$ s), only $CC_{meas}$ would change with the new sensor, increasing from 194 mAs to 348 mAs, while the value of both $CC_{sleep}$ and $CC_{upload}$ would remain the same. As a result we would obtain a consumption per cycle of 2011 mAs instead of 1237 mAs, and now the battery would last approximately 18 days, that is, about a 40% less.

We would also like to comment on some of the limitations and disadvantages of our design. In our view, the main one is the need for maintenance, i.e., the need to recharge the battery and the possible recalibration due to sensor ageing, although the latter is common to all the systems based on MOX sensors. Another aspect that could be seen as a disadvantage is the use of WiFi as a communication system, as its power consumption is quite high. However, as we said before, it also has an important advantage, as the WiFi infrastructure is available in many places and is directly usable without the need of any additional gateway.

## 4. Conclusions

In this work we showed the complete development at software and hardware level (including the development of a PCB) of a battery-operated IoT system for the measurement with TGS26xx series gas sensors by Figaro. The system uses the method of measurement in the *initial action period*, which makes it viable to be powered with batteries. This strategy allows to make the battery duration about 42 times longer (with respect to measuring using a heating period of 2–3 min as recommended by the manufacturer) in a typical use scenario, with measurements every 240 s. Therefore, with the proposed method it would be possible to power the system with a small solar cell, which would recharge the daily consumption of the battery. Besides, we kept the cost of the developed system very low, thanks to the use of common hardware and free software.

It has also been shown that storing several measurements (using the RTC memory of the SoC ESP8266EX) and uploading them as a batch to the IoT platform extends the battery duration, because the time in which the Wi-Fi modem works is reduced. However, this improvement in consumption due to measurement storage has an asymptotic behaviour, so it is not practical to accumulate more than 3 to 5 measurements.

Finally, regarding our future work we will focus on applying the methodology presented in this work to more modern sensors, with I2C digital interface, whose heater consumes less power, and we will also consider the use of lower-power wireless communications, such as Bluetooth or LoRa.

## Figures and Tables

**Table 1:** Summary of the ESP8266EX’s low-power modes.

Item	Modem-Sleep	Light-Sleep	Deep-Sleep
GPIO state	unchanged	unchanged	unchanged
Wi-Fi	OFF	OFF	OFF
System clock	ON	OFF	OFF
RTC	ON	ON	ON
CPU	ON	Pending	OFF
Substrate current	15 mA	0.4 mA	≈20 μA

**Table 2:** Summary of the elements activated at each time.

Item	Measuring	Uploading to IoT	Waiting between Measurements
ESP8266-CPU	ON	ON	Deep-Sleep
ESP8266-Wi-Fi	OFF	ON	OFF
Measurement circuit	ON	OFF	OFF
Expected consumption	≈60 mA	≈85 mA (with peaks)	≈20 μA

**Table 3:** Consumption in the “waiting between measurements” state. The real values are the average result of 15 measurements.

Jumper	Expected Current	Measured Real Value	Consumed Charge in 60 s	Consumed Charge in 120 s	Consumed Charge in 240 s
JP_PW (Global)	≈46 μA	42.8 ±0.3μA	2.57 ±0.02 mAs	5.14 ±0.04 mAs	10.27 ±0.07 mAs
JP_PW2 (Meas. Circuit)	≈0 μA	0.02 ±0.01μA	1.2 ±0.6μAs	2.4 ±1μAs	4.8 ±2μAs
JP_PW3 (ESP8266)	≈20 μA	28.6 ±0.4μA	1.72 ±0.02 mAs	3.43 ±0.05 mAs	6.9 ±0.1 mAs

**Table 4:** Consumption in “measuring-only” state. Data for different values of $t_{meas}$ are shown.

Measurement Time ($t_{meas}$)	Average Current	Average Active Time	Consumed Charge
1 s	69.3 ±0.5 mA	1.48 ±0.01 s	102.6 ±1.7 mAs
2 s	79.1 ±0.2 mA	2.44 ±0.01 s	193.9 ±0.9 mAs
3 s	84.3 ±0.3 mA	3.47 ±0.01 s	292.5 ±1.6 mAs
4 s	80.5 ±0.4 mA	4.45 ±0.01 s	358.1 ±1.7 mAs
5 s	81.5 ±0.1 mA	5.43 ±0.01 s	443.4 ±0.4 mAs

**Table 5:** Estimated battery life for several cases of interest. The capacity of the battery is considered to be 750 mAh.

Measurement Time ($t_{meas}$)	Number of Measurements ($N_{meas}$)	Deep-Sleep Time ($t_{meas}$)	Cycle Time ($t_{cycle}$)	Cycle Charge (${CC}_{cycle}$)	Battery Duration ($t_{bat}$) in Days
1	5	60 s	310.4 s	776.8 mAs	12.5
2	1	120 s	120.6 s	417.8 mAs	9.4
2	5	120 s	615.4 s	1211.8 mAs	15.9
2	5	180 s	915.4 s	1225.6 mAs	23.4
1	10	180 s	1827.6 s	2229.94 mAs	25.6
2	5	240 s	1215.4 s	1237.5 mAs	30.7
3	5	240 s	1220.4 s	1659.6 mAs	23.0
1	10	240 s	2417.4 s	1411.3 mAs	53.5

## Supplementary Materials

The following are available online at https://www.mdpi.com/1424-8220/19/14/3183/s1, Figure S1: Typical initial action of a duty cycling gas sensor (red line), versus a continuously energized one (black line), Figure S2: Image of one of the ThingSpeak IoT platform pages.

## Abbreviations

The following abbreviations are used in this manuscript:

ADC	Analog Digital Converter
GPIO	General Purpose Input Output
I2C	Inter-Integrated Circuit Bus
ICA	Independent Component Analysis
IoT	Internet of Things
JSON	JavaScript Object Notation
MEMS	Microelectromechanical systems
MOX	Metal Oxide Semiconductor
OTA	Over the Air programming
PCA	Principal Component Analysis
PCB	Printed Circuit Board
RTC	Real-Time Clock
SoC	System on Chip
SPI	Serial Peripheral Interface
SVM	Support Vector Machines
WSN	Wireless sensor network
