# Supplementary figures and images for: Low-Power and Low-Cost Environmental IoT Electronic Nose Using Initial Action Period Measurements
Source: Sensors (Basel). 2019 Jul 19;19(14):3183. doi: 10.3390/s19143183 (PMC6679561; doi:10.3390/s19143183)

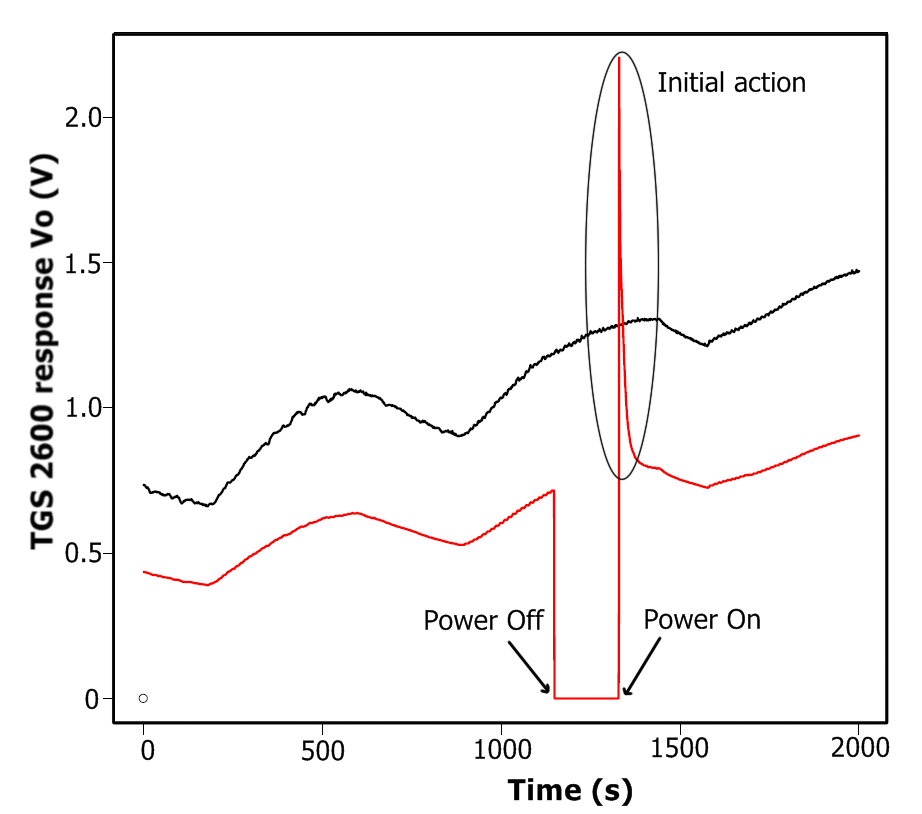

Supplement: Supplementary file 1 [file sensors-19-03183-s001.zip › Figure_S1.png]

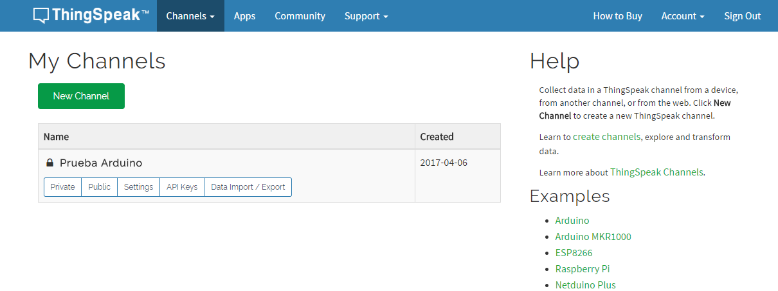

Supplement: Supplementary file 1 [file sensors-19-03183-s001.zip › Figure_S2.png]
